# Evaluating the use of paralogous protein domains to increase data availability for missense variant classification

**DOI:** 10.1186/s13073-023-01264-6

**Published:** 2023-12-12

**Authors:** Adam Colin Gunning, Caroline Fiona Wright

**Affiliations:** 1 Department of Clinical and Biomedical Sciences (Medical School, Faculty of Health and Life Sciences, University of Exeter, RILD, Barrack Road, Exeter, EX2 5DW UK; 2 Exeter Genomics Laboratory, South West Genomic Laboratory Hub, Royal Devon University Healthcare NHS Foundation Trust, RILD, Barrack Road, Exeter, EX2 5DW UK

**Keywords:** Variant classification, Missense variant, Protein domain, Bayesian, Genomic medicine

## Abstract

**Background:**

Classification of rare missense variants remains an ongoing challenge in genomic medicine. Evidence of pathogenicity is often sparse, and decisions about how to weigh different evidence classes may be subjective. We used a Bayesian variant classification framework to investigate the performance of variant co-localisation, missense constraint, and aggregating data across paralogous protein domains (“meta-domains”).

**Methods:**

We constructed a database of all possible coding single nucleotide variants in the human genome and used PFam predictions to annotate structurally-equivalent positions across protein domains. We counted the number of pathogenic and benign missense variants at these equivalent positions in the ClinVar database, calculated a regional constraint score for each meta-domain, and assessed this approach versus existing missense constraint metrics for classifying variant pathogenicity and benignity.

**Results:**

Alternative pathogenic missense variants at the same amino acid position in the same protein provide strong evidence of pathogenicity (positive likelihood ratio, LR+  = 85). Additionally, clinically annotated pathogenic or benign missense variants at equivalent positions in different proteins can provide moderate evidence of pathogenicity (LR+  = 7) or benignity (LR+  = 5), respectively. Applying these approaches sequentially (through PM5) increases sensitivity for classifying pathogenic missense variants from 27 to 41%. Missense constraint can also provide strong evidence of pathogenicity for some variants, but its absence provides no evidence of benignity.

**Conclusions:**

We propose using structurally equivalent positions across related protein domains from different genes to augment evidence for variant co-localisation when classifying novel missense variants. Additionally, we advocate adopting a numerical evidence-based approach to integrating diverse data in variant interpretation.

**Supplementary Information:**

The online version contains supplementary material available at 10.1186/s13073-023-01264-6.

## Background

The classification of sequence variants implicated in rare monogenic diseases has improved markedly since the publication of the American College of Medical Genetics and Genomics and the Association for Molecular Pathology (ACMG/AMP) guidelines in 2015 [1]. The guidelines separated evidence into different categories, including population data, computational and predictive data, functional data, genetic data (allelic, segregation and de novo), other databases (including disease and locus-specific databases), and other data (e.g. phenotype). Each line of evidence was given a particular weighting — from Very Strong (VS) and Strong (S) through to Moderate (M) and supPorting (P) — and a criterion code, which could be combined in specified ways to reach an overall variant classification ranging from benign (B) and likely benign (LB) through uncertain (U) to likely pathogenic (LP) and pathogenic (P). Since the guidelines were published, a number of supplementary papers have been published providing guidance on specific classification criteria, such as PVS1 [2], PP1 [3], PS3 [4], PP5 [5] and PP3 [6–8]. Other studies have given guidance on the combination of evidence [9–12], as well as national [13] and disease-specific [6] guidance. The aim of these publications is to make variant classification more objective, evidence-based and consistent, to ensure robust reporting of genetic results. However, the guidelines still give some room for subjectivity and a number of studies have identified inconsistencies in variant classification [14–16].

One of the biggest challenges in variant classification remains the assessment of rare missense variants that may affect protein structure, function and/or stability. Prior observation of a pathogenic missense variant at the same position in the same protein can be used as evidence of pathogenicity through the current PM5 (different missense) or PP5 (same missense) criteria, based on either literature or variant databases [17]. However, every individual has > 100 very rare missense variants in their genome [18], so in many cases a missense variant of interest will be novel. Numerous in silico pathogenicity prediction tools have been developed to aid missense variant classification, based on a variety of features that underlie pathogenicity [19–21], and evidence from these tools can be applied through a mirrored pair of variant classification codes, PP3 (supporting pathogenicity) or BP4 (supporting benignity) [6, 8]. Protein domain location and 3D structure may also be used to determine the functional importance of particular residues or hotspots [22] and the evidence applied through the PM1 criterion [23]. Missense constraint (i.e. intolerance to missense variation) can also be used as evidence through the PP2 criterion and may be gene-wide, such as the gnomAD missense-Z or observed/expected (missense_o/e) scores [24, 25] or sub-genic, such as the constrained coding regions (CCR) model [26] or MPC score [27]. Although the performance of missense pathogenicity predictors has been evaluated in the context of a Bayesian variant classification framework [8, 10], the performance of these other metrics — or their combinations — has not been formally assessed.

**Fig. 1:**
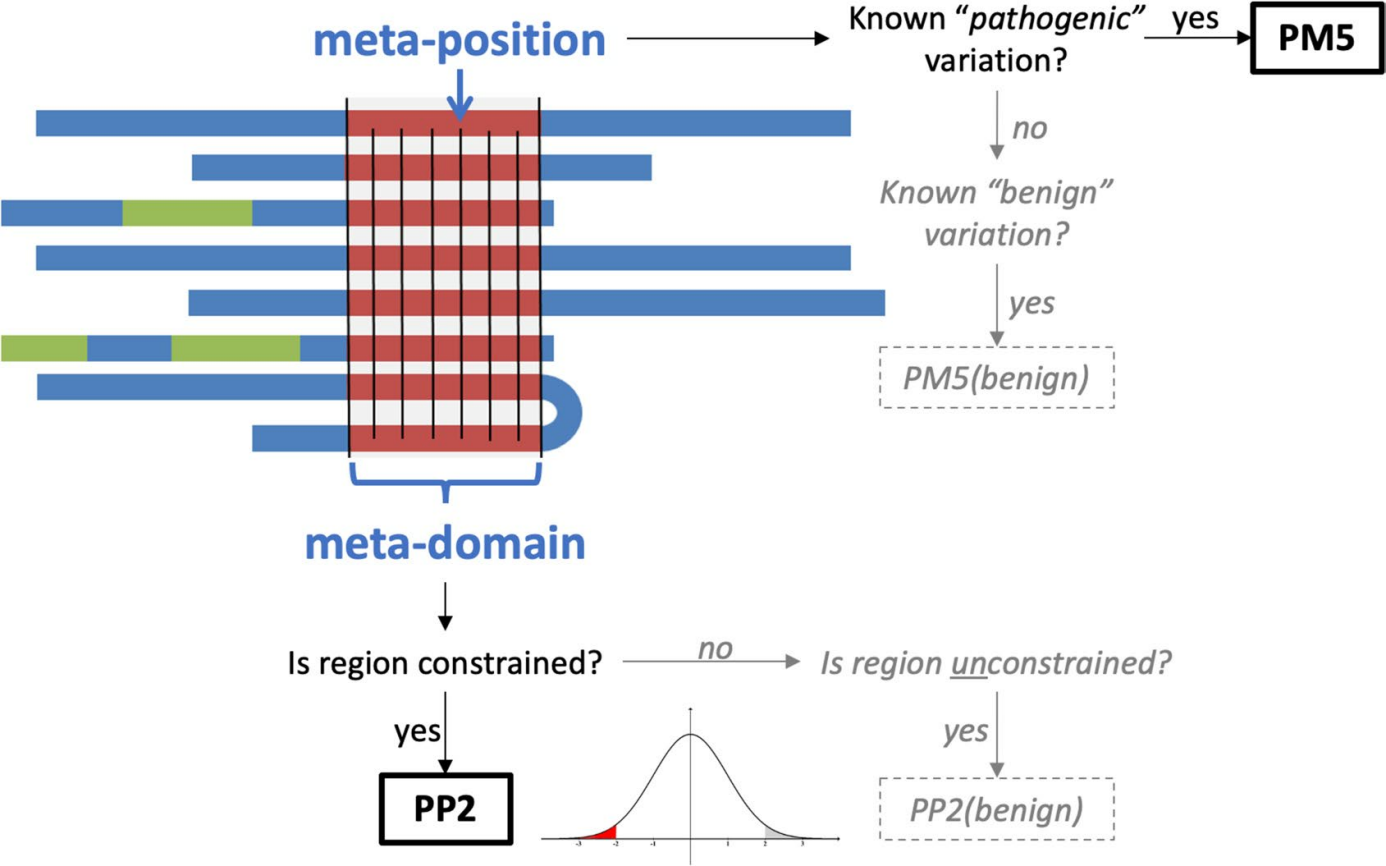
Outline of the use of meta-domain data in two variant assessment criteria (PM5 and PP2). Seven different proteins are depicted that share a common protein domain (in red). The occurrence of clinically annotated variants at a meta-position is applied under PM5, and the genetic constraint across a meta-domain is applied in PP2. The analysis also includes the use of these approaches to support the benignity of the variant (grey dotted boxes), which is not implemented in the current guidelines

 Constraint to variation has been widely used in variant prioritisation [27–29], but it is limited by the number of variants observed over a given region. This problem can potentially be overcome by aggregating data from multiple regions of the genome that are functionally equivalent — for example, paralogous protein domains [30–36]. Paralogs are homologous genes within the human genome which arose via gene duplication, whose protein products often retain overlapping functions and similar 3-dimensional protein structures. Applying information across homologous regions of DNA is not a novel idea, and can be seen as an extension of conservation analysis, which uses comparison of orthologous proteins between different species to determine sequence similarity. The equivalent approach for constraint uses aggregation of variation data across paralogous proteins across the human genome to evaluate intolerance to variation, either at a structurally equivalent “meta-position” or throughout a “meta-domain”. Here we use meta-domains to aggregate data from paralogous protein domains across the genome based on PFam domain predictions [37, 38], and assess the performance of this approach to increasing data availability against two specific variant classification criteria [1]: PM5 (“Novel missense change at an amino acid residue where a different missense change, determined to be pathogenic, has been seen before”) and PP2 (“Missense variant in a gene that has a low rate of benign missense variation and where missense variants are a common mechanism of disease”) (Fig. 1).

## Methods

### Database creation

A database containing codon-level information for every protein-coding position in the human genome was created and annotated with data from Ensembl, Uniprot, PFam, gnomAD and ClinVar. Existing annotated links between Ensembl Genes 93 and Uniprot protein sequences were accessed through Ensembl Biomart and confirmed through a direct comparison of the amino acid sequences. For further downstream annotation, only transcripts for which a link between Uniprot and Ensembl could be found were included. Exon genomic coordinates (GRCh38) from Ensembl BioMart were used to map amino acid codon positions to genomic co-ordinates by assigning each coding position in the exon to each sequential amino acid, allowing for codons to span exon/exon boundaries. The sum of the available genetic data aggregated from all paralogous protein domains, as defined by PFam, will henceforth be referred to as the “meta-domain” (Fig. 1).

To determine structurally equivalent positions across paralogous domains, protein domain annotations were added by downloading data from the PFam FTP server (date accessed: 22/06/2018) (Additional file 1: Fig. S1). The Stockholm alignment for a particular PFam domain contains a series of sequences derived from annotations of that PFam domain. The alignment contains the amino acid sequence of each domain, with ‘insertions’ and ‘deletions’, where the algorithm has predicted the insertion of an additional amino acid into the domain or a deletion of an amino acid from the domain. Once the insertions and deletions are considered the domains are all of identical length. Each position within the PFam Stockholm formatted alignments was then numbered, with deletions skipped, and insertions annotated with an incremental suffix from the preceding non-insertion position (in a similar fashion to the annotation of introns in cDNA nomenclature). The sum of the available genetic data aggregated from all functionally equivalent positions in paralogous protein domains, as defined by PFam, will henceforth be referred to as the “meta-position” (Fig. 1).

### Meta-position variant annotation

For every codon in the database, an exhaustive list of all possible single nucleotide variants (SNVs) was created by simulating the three possible nucleotide changes possible at each and every codon position, and the predicted consequence of the SNV (missense, synonymous, nonsense) annotated manually based on the amino acid change. Allele number (AN) and allele count (AC) from gnomAD v3.0 [24] (date accessed: 25/10/2019) were annotated against each simulated variant; only SNVs with a filtering annotation of PASS were selected. REVEL scores were annotated against all missense variants in the database using the dbNSFP v4.2a database [39, 40] (date accessed: 10/08/2021), which contains REVEL scores linked to GRCh38 genomic coordinates. In each case, REVEL scores were annotated using the chromosome, position (GRCh38), REF amino acid and ALT amino acid of the missense change. Variant pathogenicity in the ClinVar database [41] was annotated by downloading variants from the ClinVar FTP server [date accessed: 03/01/2022], and filtering to include only missense SNVs with unconflicting assertions of pathogenicity (P and LP) or benignity (B and LB).

### Meta-position pathogenic variant classification [PM5]

The number of ClinVar P/LP and B/LB variants at each meta-position was counted, and those with only one variant were marked as ‘unique’. Meta-positions with two or more ClinVar variants were assigned as ‘benign’ or ‘pathogenic’ if all assertions were B/LB or P/LP respectively, and ‘conflicting’ based on two alternative rules: a “no-conflict rule”, where any meta-positions with both pathogenic and benign variants were considered conflicting; and a “majority-rule”, where a meta-position was assigned to the most common pathogenicity assertion and only considered conflicting if the number of B/LB and P/LP variants was equal. A full list of variants with classifications is provided in Additional file 2: Table S1. An additional analysis including only ClinVar pathogenic variants with a REVEL score ≥ 0.7 and benign variants with a REVEL score ≤ 0.2 was performed to evaluate the value of using a pathogenicity predictor to filter variants included in the analysis. For both no-conflict and majority-rules, the performance of meta-positions for pathogenic variant classification was evaluated as follows: true positives (TP) and false positives (FP) were pathogenic and benign variants, respectively, at positions assigned as pathogenic; false negatives (FN) and true negatives (TN) were pathogenic and benign variants, respectively, at positions assigned as benign, conflicting or unique. The classifications for the contingency table are also shown in Additional file 1: Table S2. For comparison, we also assessed the performance of standard PM5 as follows: TP and FP were pathogenic and benign variants, respectively, with a different pathogenic missense variant reported at the same amino acid position in the same protein; TN and FN were benign and pathogenic variants, respectively, which had either a benign variant or no alternative variant or both pathogenic and benign variants reported at the same amino acid position in the same protein.

### Meta-position benign variant classification (PM5(benign))

Although there is no paired benign equivalent to PM5 in the current guidelines, we also wished to evaluate whether the presence of an alternative benign missense variant reported at the same position could be used in variant classification. For both no-conflict and majority-rules, the performance of meta-positions for benign variant classification was evaluated in a similar but inversed manner: TP and FP were benign and pathogenic variants, respectively, at positions assigned as benign; FN and TN were benign and pathogenic variants, respectively, at positions assigned as pathogenic, conflicting or unique. For comparison, we also assessed the performance of PM5(benign) as follows: TP and FP were benign and pathogenic variants, respectively, with a different benign missense variant reported at the same amino acid position in the same protein; TN and FN were pathogenic and benign variants, respectively, which had either a pathogenic variant or no alternative variant or both pathogenic and benign variants reported at the same amino acid position in the same protein. The classifications for the contingency table are also shown in Additional file 1: Table S3.

### Meta-domain constraint calculation

A regional meta-domain constraint score for PFam domains was created using both a raw and background-adjusted missense/synonymous (m/s) metric, as has been done previously [30]. The m/s rate (also known as d_N_/d_S_) was calculated by counting the number of missense and synonymous variants observed in the gnomAD v3.0 dataset across all instances of a PFam domain in the human genome, then adjusted for the sequence composition of surrounding regions to take account of all possible variation at each position:

$$adjusted\;m/s=\frac{\sum\left(\frac{{missense}_{obs}}{{missense}_{poss}}\right)}{\sum\left(\frac{{synonymous}_{obs}}{{synonymous}_{poss}}\right)}$$

where ‘obs’ represents the observed variation within the gnomAD database, and ‘poss’ represents the possible variation based on the sequence composition. Positional scores were aggregated across regions, and a single score was calculated for each PFam domain based on the variants observed in all members of the domain (including multiple domains in the same protein as well as domains in different proteins). PFam positions marked as ‘insertions’ were excluded. Where a genomic region was present in multiple transcripts or multiple genes, each genomic coordinate was only included in the calculation of a protein domain’s score once, but a single genomic coordinate could be included in the calculation for multiple different domains.

### Meta-domain pathogenic variant classification (PP2)

The distribution of meta-domain constraint scores was evaluated and used to determine thresholds for assigning variant pathogenicity or benignity. The thresholds for assigning pathogenicity from the meta-domain constraint score (adjusted m/s ≤ 0.34) was set as the highest score needed to reach a positive likelihood ratio (LR+) of ≥ 4.33, based on previously published thresholds required for evidence to be applied at the moderate level [10]. For comparison, we also assessed the performance of two existing gene-wide constraint measures (gnomAD missense_Z and missense_o/e) [24, 25] and a single regional constraint score (Constraint Coding Regions, CCR) [26], using the recommended thresholds for assignment of pathogenicity. The performance of constraint for pathogenic variant classification was evaluated as follows: TP and FP were pathogenic and benign variants, respectively, that met or exceeded the set pathogenic threshold; FN and TN were pathogenic and benign variants, respectively, that did not meet the tool-specific threshold.

### Meta-domain benign variant classification (PP2(benign))

We also wished to evaluate whether the absence of constraint could be used to assess benignity. Since there is no paired benign equivalent to PP2 in the current guidelines, thresholds for assigning benignity were set to give a similar sensitivity as the standard pathogenic analysis, i.e. by selecting a score at the same number of standard deviations from the mean as the pathogenic threshold. This could be done for all tools except CCR, which gives scores as a centile rank in a bimodal distribution, so the lower threshold was simply set to the high threshold subtracted from 100. The performance of constraint for benign variant classification was evaluated as follows: TP and FP were benign and pathogenic variants, respectively, that met or exceeded the set benign threshold; FN and TN were benign and pathogenic variants, respectively, that did not meet the tool-specific threshold.

## Results

### Alternative pathogenic missense variants at the same amino acid position in the same protein provide strong evidence of pathogenicity

Across 37,648 pathogenic and 49,122 benign missense variants included in our analysis, 86% were unique, i.e. had no other variant at the same amino acid position in the same protein. In non-unique positions, we found that the standard PM5 analysis had a sensitivity of 0.274 and a positive likelihood ratio of 85 (Table 1), consistent with the evidence being applied with a strong weighting (LR+  ≥ 18.71) rather than the moderate weighting (LR+  ≥ 4.33) originally suggested in the current guidelines [1, 10]. When ClinVar variants were additionally filtered according to their REVEL scores (with pathogenic variants only being counted with a REVEL score ≥ 0.7, and benign variants only being counted with a REVEL score ≤ 0.2), the proportion of unique variants increased to 89% and the positive likelihood ratio for pathogenicity at non-unique positions increased to 148.

**Table 1:**
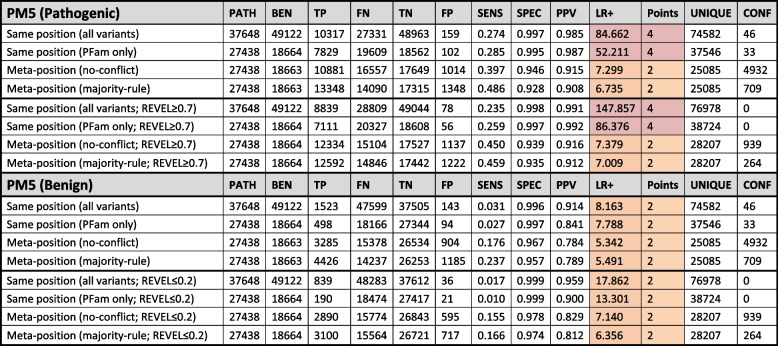
Performance of PM5 pathogenic (top) and benign (bottom) analysis for co-localising clinically annotated variants

Shading in the LR+ and points columns indicates the strength reached for classification purposes according to Tavtigian et al. (2018) and Tavtigian et al. (2020) (red = strong; amber = moderate). *PFam only* ClinVar missense variants in PFam domains, *PATH* Number of pathogenic variants, *BEN* Number of benign variants, *TP* True positive, *FN* False negative, *TN* True negative, *FP* False positive, *SENS* Sensitivity, *SPEC* Specificity, *PPV* Positive predictive value, *LR*+  Positive likelihood ratio, *UNIQUE* Number of position with only a single variant or no co-localising variants, *CONF* Positions with conflicting classifications

### Clinically annotated pathogenic or benign missense variants at the equivalent domain position in different proteins provide moderate evidence of pathogenicity or benignity respectively

Restricting our analysis to ClinVar variants located within PFam domains resulted in a reduction to 27,438 (73%) pathogenic and 18,664 (38%) benign variants, consistent with a significant enrichment of pathogenic versus benign missense variants in protein domains versus outside domain regions (chi-squared *P* < 0.0001). The inclusion of additional data from structurally equivalent meta-positions across different proteins substantially decreased the proportion of variants that were unique to 54% and increased the sensitivity of the analysis to 0.397 (no-conflict) or 0.486 (majority-rule) whilst slightly decreased the specificity to 0.946 (no-conflict) and 0.928 (majority-rule) (Table 1). The positive likelihood ratio also decreased to around 7, consistent with the evidence being applied with a moderate weighting under PM5.

Interestingly, all PM5(benign) analyses resulted in positive likelihood ratios consistent with evidence for benignity being applied at the moderate level (Table 1). As with the standard pathogenicity analysis, the use of meta-domains greatly increased the sensitivity (from 0.027 in the standard analysis to 0.237 using the meta-position majority-rule approach) and slightly decreased the specificity (from 0.997 to 0.957). None of the results changed substantively when variants were additionally filtered according to the REVEL scores, though the number of conflicting positions decreased substantially.

### Missense constraint can provide strong evidence of pathogenicity in a small proportion of variants, but the absence of constraint provides no evidence of benignity

**Fig. 2:**
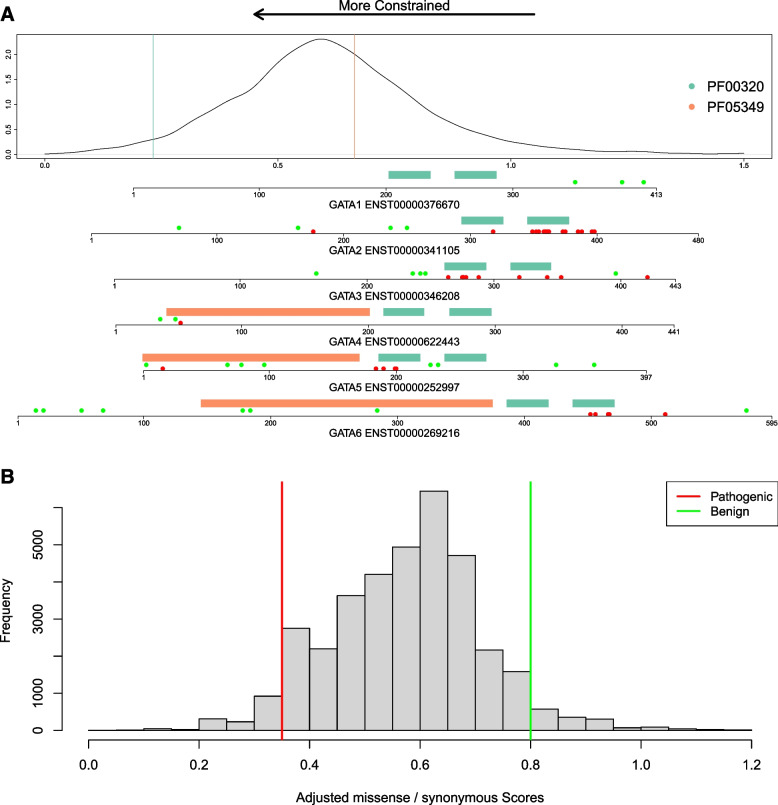
Score distribution and thresholds for meta-domain constraint. **a** Example of domain structure and meta-domain constraint for six related proteins, GATA1-6. The density plot shows the distribution of the adjusted m/s constraint scores for all PFam domains, and the coloured vertical lines correspond to the specific domains within the GATA1-6 proteins (GATA zinc finger domain, PF00320 = green; GATA-type transcription activator N-terminal domain, PF05349 = orange), all of which have been implicated in monogenic diseases. Below the density plot, a separate (N-terminal aligned) plot is shown for each protein, showing locations of the PFam domains (coloured as above), and ClinVar benign/likely benign (green) and pathogenic/likely pathogenic variants (red).** b** Histogram showing the distribution of adjusted m/s scores in the ClinVar pathogenic and benign missense variant dataset. Vertical lines indicate pathogenic (red) and benign (green) thresholds for the PP2 analysis

An example of the meta-domain constraint scores is provided for six related proteins (GATA1-6) containing two paralogous domains (Fig. 2a). Notably, ClinVar pathogenic variants cluster around the more constrained domains (PF00320), whilst benign variants cluster around the less constrained domains (PF05349). Across all PFam domains, meta-domain constraint values are approximately normally distributed, allowing us to select thresholds for variant classification across all domains for comparison with alternative constraint metrics (Fig. 2b).

**Fig. 3:**
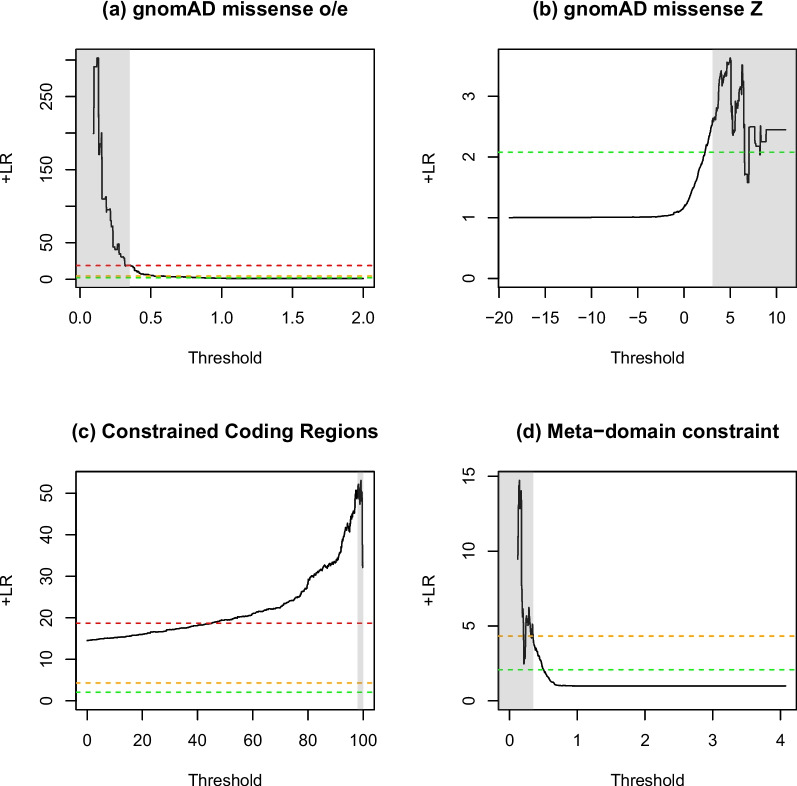
Positive likelihood ratio curves for constraint metrics. The effect on the positive likelihood ratio (*y*-axis) based on the tool threshold (*x*-axis) for **a** gnomAD missense_oe, **b** gnomAD missense_Z,** c** small constrained coding regions (CCR) and **d** adjusted m/s meta-domain constraint. Each analysis was done 1000 times with equal steps between the minimum and maximum values. Vertical shading indicates tool-specific thresholds for pathogenicity (see Table 2) and horizontal dotted lines indicate likelihood ratio thresholds for pathogenic variant classification from Tavtigian et al. (2018) (green = supporting, amber = moderate, red = strong)

For existing gnomAD gene-wide constraint metrics [24, 25], missense_o/e outperforms missense_Z, achieving a positive likelihood ratio of 19.060 but with a low sensitivity (0.032), which is consistent with the evidence being applied with a strong weighting under PP2, versus 2.568, which is only consistent with evidence being applied at supporting (LR+  ≥ 2.08) (Table 2). The published regional constraint measure, CCR [26], performs better than missense_o/e with a positive likelihood ratio of 49.121, again consistent with the evidence being applied with a strong weighting, but with a low sensitivity (0.025) due to the small coverage of CCRs. The very high specificity and low sensitivity of the missense_o/e and CCR scores indicate that the recommended thresholds for these tools may be too stringent, and a better balance between sensitivity and specificity could potentially be achieved using a lower threshold — particularly for CCR — whilst still meeting the LR+ requirements for classification purposes (Fig. 3). The new meta-domain constraint score (adjusted m/s) performs comparably, with positive likelihood ratios of 4.326 and 4.583, respectively, consistent with a moderate evidence weighting.

**Table 2:**
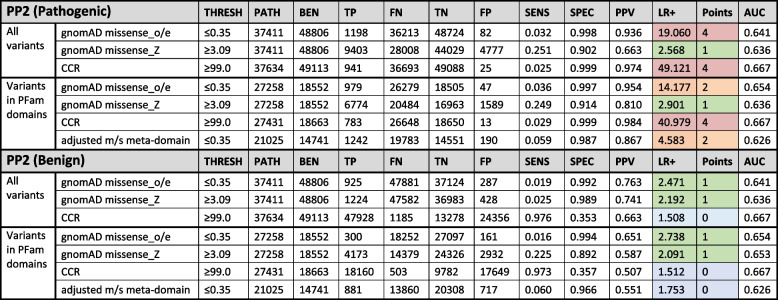
Performance of PP2 pathogenic (top) and benign (bottom) analysis for five genetic constraint metrics

Shading in the LR+ and points columns indicates the strength reached for classification purposes according to Tavtigian et al. (2018) and Tavtigian et al. (2020) (red = strong; amber = moderate; green = supporting; blue = below minimum evidence weighting). *gnomAD missense_o/e*, observed/expected missense variants from gnomAD, *CCR* Constrained coding regions from Havrilla et al. (2019), *THRESH* Tool-specific threshold, *PATH* Number of pathogenic variants, *BEN* Number of benign variants, *TP* True positive, *FN* False negative, *TN* True negative, *FP* False positive, *SENS* Sensitivity, *SPEC* Specificity, *PPV* Positive predictive value, *LR*+  Positive likelihood ratio, *AUC* Area under the ROC curve (calculated in R using the pROC library)

The analysis of using missense constraint as evidence of benignity indicated that none of the tools performs well (Table 2), with only gnomAD missense_o/e and missense_Z reaching a positive likelihood ratio sufficient to be used even as supporting evidence (2.471 and 2.192, respectively), with very low sensitivities of 0.019 and 0.025, respectively.

## Discussion

We have assessed the performance of two related aspects of variant classification, co-localised clinically annotated variation (PM5) and genetic constraint (PP2), as evidence for either pathogenicity or benignity using a Bayesian framework [8, 10]. We further extended the analysis to assess the benefit of aggregating data across equivalent domains in different proteins, using structurally equivalent meta-positions to augment variant co-occurrence and meta-domains to calculate regional constraints.

**Fig. 4:**
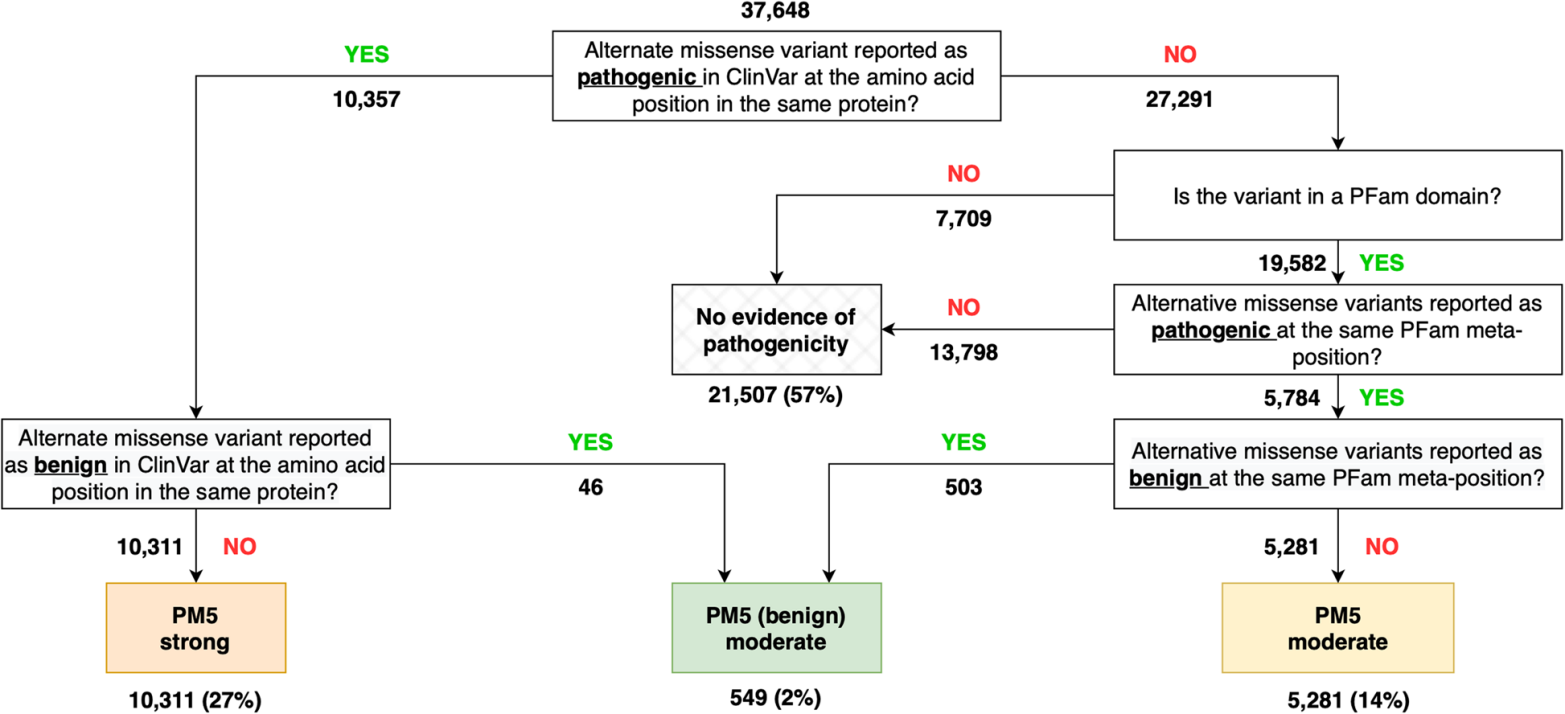
Flow diagram of the proposed logic for applying variant co-localisation data. Standard PM5 based on variants at the same position in the same protein (left) is augmented by variants at equivalent meta-positions in domains across different proteins (right). Numbers represent the number of variants at each step if the logic were applied to the pathogenic variants selected for this study. The analysis allows for the classification of an additional 5281 variants missed by the standard analysis, increasing the sensitivity from 0.27 to 0.41

We show that the presence of an alternative pathogenic missense variant at the same position in the same protein provides strong evidence of pathogenicity and that a pathogenic missense variant at the same meta-position in a different protein provides moderate evidence of pathogenicity. We suggest that these approaches could be combined using a cascading approach (Fig. 4), which would allow PM5 to be applied at a reduced weighting to an additional 14% of variants for which the standard PM5 analysis is not applicable, increasing the sensitivity from 0.27 to 0.41. Consistent with other studies [42], we also note the higher likelihood ratio when restricting ClinVar variants to those with high REVEL scores, further supporting the graded use of PM5. It should be noted that there was no manual classification of ClinVar variants in our analysis, which is contrary to the current stipulations of the guidelines specifying that variants at the same position must be classified manually and established to be pathogenic. However, our results suggest that this onerous manual classification step is not necessarily required, as the evidence already performs above the level implemented within the current framework; the performance of this approach without the need for manually variant classification suggests that it could form part of a wider in silico approach using a machine-learning classification framework.

We further show that two constraint metrics (gnomAD missense_o/e and CCR) provide strong evidence of pathogenicity, with the CCR providing the best performance across the different approaches taken to calculating constraint, albeit for a very small number of variants. As before, there is a trade-off between sensitivity and precision, and a cascade approach could maximise the utility of the data — using small regional approaches to constraint where possible [43], followed by meta-domain or gene-level constraint. The lower performance of the meta-domain constraint metrics is likely due to the aggregation of data across a range of genes, only some of which are linked with disease, which would potentially be improved by limiting to conserved positions. Although within-species constraint and inter-species conservation are highly correlated, they are derived using orthogonal datasets and thus can be treated as independent sources of evidence and used additively in variant classification. Moreover, although domains potentially provide evolutionarily informed regions over which to aggregate human variation data and calculate constraint, combining data across proteins that are under very different evolutionary pressures will necessarily result in reversion to the mean. The methodology also assumes a functional link between amino acids at the same structural position within a domain, which is not always true across proteins that perform different functions. Any approach that aggregates data over protein domains is necessarily limited to the ~ 31% of amino acids of the human proteome predicted to lie within known structured protein domains, and therefore the results are not amenable to every variant. Nonetheless, genetic constraint at meta-positions has been shown to outperform other variant prioritisation methods [36], and meta-domains have potential utility for aiding the interpretation of variants in related families of genes associated with disease by highlighting where variation is less well tolerated (see example in Fig. 2a).

The lack of benign sources of evidence is a potential weakness of the current variant classification guidelines. In order to implement a truly Bayesian approach, it must be possible for evidence to support the benignity of a variant as well as its pathogenicity. This may be more complicated than simply implementing the negative likelihood ratio from an analysis, especially where intermediary ranges are present and the pathogenic and benign methodologies are implemented separately. We therefore evaluated the potential for co-localising benign variants and lack of constraint (due to higher-than-expected missense variation) to be used as evidence for benignity. In both cases, the benign analysis performed less well than the pathogenic analysis, which may in part be explained by the relative depletion of benign variants within domains. Nonetheless, PM5(benign) reached positive likelihood ratios consistent with moderate evidence levels and may therefore be a useful addition to variant classification (Fig. 4). However, we suggest that this evidence should only be applied with caution. The presence of benign missense variation at a particular position cannot be taken to indicate that a pathogenic missense variant could not occur at the same location. Most notably, lack of constraint cannot be used as evidence to support benignity.

The limitations of each of the methodologies examined here are varied and must be considered when applying the evidence through variant classification. Most importantly, each of the pieces of evidence analysed here was assessed in isolation, and yet may draw heavily on other evidence sources. Each of the evidence criteria in the guidelines has been assessed under the assumption that they are completely independent data sources, as is necessary for application in a Bayesian framework. This is known to be a fallacy, and often the evidence used is highly circular, relying on similar sources. For example, all of the constraint algorithms draw their datasets from gnomAD, which is also implemented in the guidelines through the PM2 criterion. If the constraint is considered on a spectrum, with PM2 essentially being a base-level constraint measure, through regional constraint and gene-wide constraint, it follows that the regional constraint measures will show more circularity with the PM2 metric, with smaller regions being more affected. Whilst it could be posited that the meta-constraint scores will be equally affected by this circularity, by drawing on many regions of the genome considered functionally equivalent, the effects of any individual variant present within the gnomAD dataset will be diluted linearly with the number and size of the regions being assessed. Further work is needed to evaluate and address this circularity.

Another limitation is that the meta-domain approach relies on domain predictions that may be incorrect or incomplete. PFam predictions rely on sequence-based data, which may insufficiently capture the complete diversity of proteins, potentially omitting the identification of novel protein families or domains with low sequence homology. Additionally, the accuracy of PFam’s predictions can exhibit variability, contingent upon the specific protein families under examination and the availability of high-quality reference sequences, and the predictions may be less reliable for divergent or inadequately characterised protein families. Whilst other domain predictions are likely to suffer from the same issues, the limitation could potentially be addressed by using 3-dimensional protein structures, such as those predicted by AlphaFold [44–46].

Finally, the use of ClinVar variants for benchmarking is a potential weakness in our method, due to classification errors in the database. We attempted to minimise these errors by excluding variants with uncertain or conflicting interpretations, but would welcome the development of large truth sets of pathogenic and benign variants for further assessment of variant classification approaches.

## Conclusions

We advocate using an objective approach to evaluating evidence used in variant classification, that weighs the value of different types of data in an evidence-based manner. Within the context of the current ACMG/AMP variant classification guidelines [1], our analysis suggests that the standard PM5 criterion can be applied as strong evidence if a co-localised alternative missense variant in the same protein has been reported as pathogenic or likely pathogenic (with no conflicting reports). We further suggest that, where this is not available, PM5 could be applied as moderate evidence where a pathogenic missense variant has been reported at a functionally equivalent position in the same domain of a different protein. However, given the large differences in likelihood ratios between different analyses, we hope that future guidelines will take a more numerical and explicitly Bayesian approach to the use of evidence in variant classification. A methodology whereby evidence for pathogenicity or benignity could be applied directly (i.e. by combining likelihood ratios with a prior probability to calculate a posterior probability of variant pathogenicity) would provide a more evidence-based approach to variant classification, and remove thresholding effects whereby very minor changes in scores can have major impacts on variant assessment.

### Supplementary Information

**Additional file 1: Figure S1. **Pfam Stockholm Alignment and residue numbering.** Table S2. **Examples of meta-position classification.** Table S3. **Classification of variants for the PM5 and PM5(benign).

**Additional file 2: Table S1. **Full list of ClinVar variants with classifications.

## Data Availability

The full database is available on Zenodo https://zenodo.org/doi/10.5281/zenodo.10159779.
