# Supplementary material for: Evaluating the use of paralogous protein domains to increase data availability for missense variant classification
Source: Genome Med. 2023 Dec 12;15:110. doi: 10.1186/s13073-023-01264-6 (PMC10714540; doi:10.1186/s13073-023-01264-6)
Supplement: Supplementary file 1 — Additional file 1: Figure S1. Pfam Stockholm Alignment and residue numbering. Table S2. Examples of meta-position classification. Table S3. Classification of variants for the PM5 and PM5(benign). [file 13073_2023_1264_MOESM1_ESM.docx]

**Evaluating the use of paralogous protein domains to increase data availability for missense variant classification**

Gunning AC^1,2^ & Wright CF^1*^

^1^ Department of Clinical and Biomedical Sciences (Medical School), Faculty of Health and Life Sciences, University of Exeter, RILD, Barrack Road, Exeter EX2 5DW

^2^ Exeter Genomics Laboratory, South West Genomic Laboratory Hub, Royal Devon University Healthcare NHS Foundation Trust, RILD, Barrack Road Exeter UK, EX2 5DW

* caroline.wright@exeter.ac.uk

**SUPPLEMENTAL FIGURES**


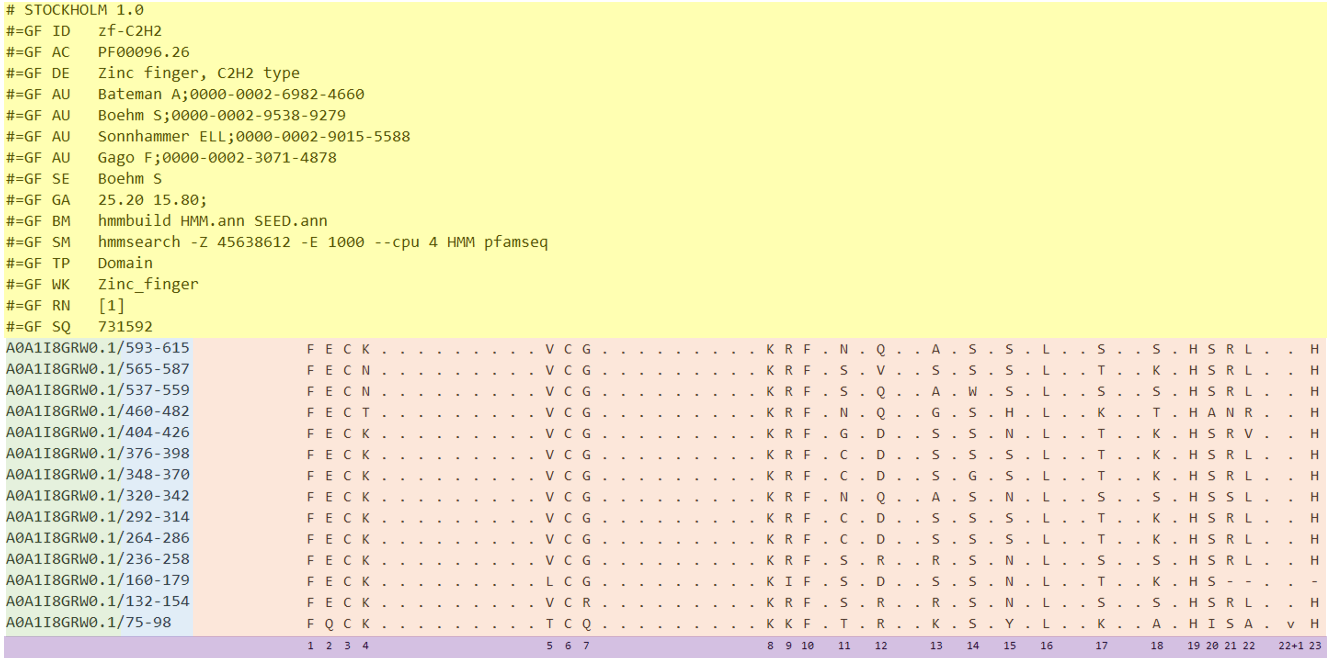


***Figure S1. Pfam Stockholm Alignment and residue numbering.***

The PFam Stockholm alignment for C2H2 zinc finger domain [PFam accession PF00096] with header information [yellow], uniprot accession [green], domain position[blue] and the multiple sequence alignment (MSA) [orange]. In the MSA, a period (“.”) represents an insertion in another domain member sequence, a dash (“-“) represents a deletion in this domain sequence, and a lowercase letter represents an insertion in this domain. The numbering [purple] shows the PFam position assignment representing the last member of the MSA (A0A1I8GRW0.1/75-98). Insertions (eg. 22+1 in the example above) are amino acids within the contiguous boundaries of the domain, but were not considered part of the domain for functional equivalence.

**SUPPLEMENTAL TABLES**

| **Meta position** | **ClinVar Variation** | | **Meta-position classification** |
| --- | --- | --- | --- |
|  | **ClinVar variants at position** | **ClinVar variant classification** |  |
| PF00096_3 | NM_014795.4:c.3170G>A (p.Cys1057Tyr)  NM_014795.4:c.3169T>C (p.Cys1057Arg)  NM_014795.4:c.851G>A (p.Cys284Tyr)  NM_001348800.3:c.1822T>C (p.Cys608Arg)  NM_014797.3:c.1222T>G (p.Cys408Gly)  NM_001320669.3:c.730T>C (p.Cys244Arg) | Pathogenic  Likely pathogenic  Likely pathogenic  Likely pathogenic  Pathogenic  Likely pathogenic | PATHOGENIC |
| PF00008_25 | NM_198576.4:c.4073C>T (p.Pro1358Leu)  NM_024408.4:c.2585C>T (p.Ala862Val)  NM_201253.3:c.866C>T (p.Thr289Met)  NM_017617.5:c.3767C>T (p.Pro1256Leu)  NM_017617.5:c.3401A>G (p.Gln1134Arg)  NM_017617.5:c.2339G>A (p.Arg780Gln)  NM_138573.4:c.109G>A (p.Val37Ile)  NM_000435.3:c.1487C>T (p.Pro496Leu)  NM_000214.3:c.1655C>T (p.Pro552Leu) | Likely benign  Benign  Likely benign  Benign/Likely benign  Benign/Likely benign  Likely benign  Benign  Benign/Likely benign  Benign/Likely benign | BENIGN |
| PF01094_48 | NM_000388.4:c.346G>A (p.Ala116Thr) | Pathogenic | UNIQUE |
| PF01094_286 | NM_000388.4:c.1285C>T (p.His429Tyr) | Benign/Likely benign | UNIQUE |
| PF00400_35 | NM_002074.5:c.239T>C (p.Ile80Thr)  NM_002074.5:c.239T>A (p.Ile80Asn)  NM_002074.5:c.239T>G (p.Ile80Ser)  NM_004766.3:c.760C>T (p.Arg254Cys)  NM_024535.5:c.578G>A (p.Arg193Gln)  NM_001083961.2:c.1312C>T (p.Arg438Cys)  NM_001083961.2:c.1313G>A (p.Arg438His) | Pathogenic/Likely pathogenic  Pathogenic  Pathogenic  Pathogenic  Benign  Likely pathogenic  Pathogenic | CONFLICTING  [no-conflict]  PATHOGENIC  [majority-rule] |

**Table S2: Examples of meta-position classification.**

| **Meta-position** | **PM5 (Pathogenic)** | | **PM5 (Benign)** | |
| --- | --- | --- | --- | --- |
|  | **Pathogenic variant** | **Benign Variant** | **Pathogenic variant** | **Benign Variant** |
| **PATHOGENIC** | True positive | False positive | True negative | False negative |
| **BENIGN** | False negative | True negative | False positive | True positive |
| **UNIQUE** | False negative | True negative | True negative | False negative |
| **CONFLICTING** | False negative | True negative | True negative | False negative |

**Table S3: Classification of variants for the PM5 and PM5(benign) [Table 1].**
